# The Impact of Selection, Gene Conversion, and Biased Sampling on the Assessment of Microbial Demography

**DOI:** 10.1093/molbev/msw048

**Published:** 2016-03-01

**Authors:** Marguerite Lapierre, Camille Blin, Amaury Lambert, Guillaume Achaz, Eduardo P. C. Rocha

**Affiliations:** ^1^Atelier de Bioinformatique, UMR7205 ISYEB, MNHN-UPMC-CNRS-EPHE, Muséum National d’Histoire Naturelle, Paris, France; ^2^Collège de France, Center for Interdisciplinary Research in Biology (CIRB), CNRS UMR 7241, Paris, France; ^3^Sorbonne Universités, UPMC Univ Paris06, IFD, 4 Place Jussieu, Paris Cedex05, France; ^4^Institut Pasteur, Microbial Evolutionary Genomics, Paris, France; ^5^CNRS, UMR3525, Paris, France; ^6^UPMC Univ Paris 06, Laboratoire de Probabilités et Modèles Aléatoires (LPMA), CNRS UMR 7599, Paris, France

**Keywords:** bacteria, population size, natural selection, gene conversion, *Escherichia coli*, population genomics

## Abstract

Recent studies have linked demographic changes and epidemiological patterns in bacterial populations using coalescent-based approaches. We identified 26 studies using skyline plots and found that 21 inferred overall population expansion. This surprising result led us to analyze the impact of natural selection, recombination (gene conversion), and sampling biases on demographic inference using skyline plots and site frequency spectra (SFS). Forward simulations based on biologically relevant parameters from *Escherichia coli* populations showed that theoretical arguments on the detrimental impact of recombination and especially natural selection on the reconstructed genealogies cannot be ignored in practice. In fact, both processes systematically lead to spurious interpretations of population expansion in skyline plots (and in SFS for selection). Weak purifying selection, and especially positive selection, had important effects on skyline plots, showing patterns akin to those of population expansions. State-of-the-art techniques to remove recombination further amplified these biases. We simulated three common sampling biases in microbiological research: uniform, clustered, and mixed sampling. Alone, or together with recombination and selection, they further mislead demographic inferences producing almost any possible skyline shape or SFS. Interestingly, sampling sub-populations also affected skyline plots and SFS, because the coalescent rates of populations and their sub-populations had different distributions. This study suggests that extreme caution is needed to infer demographic changes solely based on reconstructed genealogies. We suggest that the development of novel sampling strategies and the joint analyzes of diverse population genetic methods are strictly necessary to estimate demographic changes in populations where selection, recombination, and biased sampling are present.

## Introduction

Bacterial populations show extensive demographic variations across space and time (Martiny et al. 2006), such as frequent expansions and bottlenecks. The characterization of these demographic changes among populations of infectious agents provides epidemiological information that can guide public health interventions. A recent field of research, phylodynamics, aims at understanding the association between ecological processes and epidemiological patterns in an evolutionary framework (Grenfell et al. 2004). It integrates phylogenic inference and population genetics to study variations in demography through time (Grad and Lipsitch 2014; Li et al. 2014). Phylodynamics has been particularly useful to characterize transmission dynamics from sequence data, and could facilitate the evaluation of public health policies for diseases with low reporting rates (Volz et al. 2013).

Demographic changes imprint the reconstructed genealogies of the population, the so-called coalescent tree, by affecting the intervals of time between successive splits in the tree (Tajima 1989a). These values (coalescent rates) are proportional to the inverse of the effective population size (*N*_e_) in the standard neutral model. If one takes two idealized populations with the same contemporary population size, then the one with a history of population expansion will have (on average) shorter branches throughout, including at the tips. However, the relative length of the tips compared with the internal branches will be longer than in a nonexpanding population. Since nodes in the reconstructed genealogy of the expanding population are more concentrated closer to the root of the tree, the site frequency spectrum (SFS), that is, the distribution of the frequencies of all nucleotide polymorphisms, shows an excess of alleles shared by few individuals (rare alleles) (Adams and Hudson 2004). Conversely, populations with a history of population size contraction exhibit an excess of polymorphism shared by many individuals when compared with stable populations with the same contemporary population size. Their reconstructed genealogies have longer branches overall, but the average length of the tips compared with the internal branches are shorter than in a noncontracting population (coalescence rates are higher than expected closer to the present).

Under the assumptions of the standard neutral model (no population structure, random sampling, no recombination, no selection), it is often implicitly assumed that variations in *N*_e_ (or equivalently, variations in the coalescence rate) are indications of demographic changes. Parametric approaches were developed to infer these demographic changes under explicit models, such as the Approximate Bayesian Computation method (Beaumont et al. 2002) or the likelihood-based method (*e.g.*, Nielsen and Wakeley 2001; Drummond et al. 2002). In this context, skyline plots were introduced to quantify the relationship between the coalescence rate of the population and the genealogy of the sequences in a non-parametric approach, that is, without an explicit model to test. Coalescent rates can then be used to produce detailed demographic histories from sequence data assuming that all other assumptions of the neutral coalescent are met (Pybus et al. 2000; Drummond et al. 2005). Demographic trends can also be inferred using SFS-based neutrality tests (Fu 1997; Fu and Voordouw 1997; Ramos-Onsins and Rozas 2002; Achaz 2009). For example, Tajima’s *D* measures the difference between the mean number of pairwise differences and the number of segregating sites, and is skewed to negative values in case of population expansion (Tajima 1989b). SFS-based model-flexible methods (*i.e.*, exploring the space of possible demographic models) have also been recently proposed (Liu and Fu 2015). They approximate the demography using piecewise constant population sizes.

Violations of the assumptions of the neutral coalescent, such as presence of recombination or selection, may affect reconstructed genealogies and SFS in ways resembling demography (*e.g.*, Schierup and Hein 2000; Nielsen and Beaumont 2009; Mazet et al. 2015). Recombination by gene conversion has a very moderate effect on the topology of phylogenetic trees (Touchon et al. 2009), but affects skyline models (Hedge and Wilson 2014). Removing sites incompatible with the tree topology, that is, homoplasies, actually aggravates the effect of recombination in skyline models, presumably because it preferentially removes polymorphisms in deeper branches of the tree (Hedge and Wilson 2014). Recombination in the absence of selection has actually little effect on the expected SFS, apart from decreasing its variance (Wall 1999). The effect of selection on skyline plots has been less studied. Strong purifying selection is not expected to affect drastically the SFS because the deleterious mutations are quickly purged (Kimura 1983). On the other hand, mild purifying selection or recent selective sweeps lead to an excess of recent polymorphism, creating the impression of recent population expansion (Braverman et al. 1995). Diversifying or balancing selection can produce more complex patterns (Navarro and Barton 2002). Some studies have found that deleterious mutations of mild effect have a negligible effect on the time back to the most recent common ancestor (TMRCA) (Neuhauser and Krone 1997), and very little effect on the shape of the reconstructed genealogies (Przeworski et al. 1999) even though linkage between sites may affect the distribution of mutations (Williamson and Orive 2002). Mutations of mild deleterious effect are abundant in some bacteria (Hughes 2005; Balbi et al. 2009). If bacterial evolution is dominated by these mutations then selection might not strongly affect demographic inference using skyline plots. However, recent studies have suggested that weak purifying selection, when occurring at multiple sites, could affect the shape of the coalescent tree (O’Fallon et al. 2010). The effect of selection on skyline plots remains unclear.

The possibility of producing large sequence datasets for microbial populations has spurred interest on the use of these methods to study microbial demography. The skyline plot has been particularly popular because it allows precisely detailing demographic changes (Ho and Shapiro 2011). This method was initially used to study RNA viruses, which exhibit low recombination rates between individuals in different hosts and small effective population sizes (Holmes 2007). These viruses also have very high mutation rates, which increases mutational load and decreases the efficiency of selection (especially under no recombination) (Kimura 1983). Skyline plots have been increasingly used to study cellular microbes, most notably pathogenic bacteria. Yet, it is unclear if violations to the neutral coalescent model (biased sampling, selection, or recombination) can be safely ignored in these cases. Many bacterial populations are extremely large, show a very strong imprint of natural selection, endure rapid population fluctuations, exhibit low mutation rates, and recombine at high rates (Rocha et al. 2006; Vos and Didelot 2009; Tellier and Lemaire 2014). In fact, abundant evidence suggests that there are few, if any, positions evolving according to the neutral model in bacterial genomes (reviewed in Rocha and Feil 2010).

Most demographic analyses assume random sampling. However, sampling is usually not random in microbial studies, either on purpose or by the intrinsic difficulties of defining appropriate sampling strategies in microbiology, and this may severely affect the conclusions taken from the analysis of reconstructed genealogies. There are three major sampling biases in microbiology. *Clustered* sampling occurs when all samples are taken from a single sub-population, for example, a particularly virulent lineage. *Uniform* sampling of all major lineages is frequently found in studies aiming at maximizing the genetic diversity of samples. This bias may also result from sampling different environments (or patients) while analyzing a single isolate per site (thus disregarding differences in population sizes in each site). Finally, a very common type of *mixed* sampling bias is found in studies extensively sampling a sub-population and a small number of very diverse individuals from other sub-populations. This gives a broad view of the genetic diversity in the species, while focusing in a sub-population of interest. Analyses using sequences available in databanks are prone to combine the sampling biases of the different underlying studies.

We surveyed the available literature on the use of skyline plots to describe bacterial population demography and found that nearly all studies showed skyline plots suggestive of population expansion. We then decided to test if the violations of the assumptions of the neutral coalescent could be reasonably ignored when studying bacterial populations. For this, we simulated the evolution of bacterial populations of constant size using biologically realistic parameters for natural selection, recombination, and sampling bias. These sequences were then used to build skyline plots and make SFS-based inference of demographic changes. We did not use time calibration in the inference of the skyline plots. Therefore, the *Y*-axis in the skyline plots represents the inferred product of *N*_e_ by the mutation rate *u* (*N*_e_.*u*) and the *X*-axis represents the expected number of mutations per site, which is an estimate of the distance from the present (Ho and Shapiro 2011). By convention, we represent zero mutations per site at the left of the skyline plots. Hence, the *X*-axes of the skyline plots are ordered from the present (left) to the past (right). In the last section, we present the analysis of data from *Escherichia* *coli* in the light of the results of simulations.

## Results

### The Puzzling Expansion of Most Bacterial Populations

We found 26 recent studies using skyline plots to analyze bacterial demography. We analyzed their characteristics in terms of TMRCA, demographic changes, and their presumed justifications (table 1). The TMRCA of these populations was extremely variable, from 3 years to over 100 million years. Many of these studies proposed some type of justification for the observed demographic changes. For example, demographic expansion in *Bordetella* *pertussis* was associated with the introduction of vaccination and expansion of escape variants (Bart et al. 2014). Demographic expansion in *Clostridium difficile* was associated with the date when the bacterium became a recognized nosocomial pathogen (He et al. 2010), and in *Salmonella enterica* serovar Typhi with the introduction of antibiotics (Roumagnac et al. 2006). Skyline plots suggested that the effective population size of *Neisseria gonorrhoeae* in Baltimore increased during most of the twentieth century and then decreased, presumably as the result of urban planning and changes in patterns of drug addiction (Perez-Losada et al. 2007). Some works suggested associations between the increase in effective population sizes and environmental changes, for example, glacial cycles in *Thiomonas* spp. (Liao and Huang 2012), and human population growth in *Mycobacterium tuberculosis* (Comas et al. 2013). However, a careful analysis of table 1 revealed a most puzzling trend: the vast majority of studies (21 out of 26) concluded that effective population sizes have increased.

**Table 1.** Published Works Using Skyline Plots to Estimate Demographic Changes in Bacteria.

Species	Conclusion	TMRCA	Authors’ Comments
*Bordetella pertussis*	Expansion	200 Y	Surprisingly, vaccination was followed by increase not decrease in *N*_e_.*u*, suggesting diversification of lineages escaping the vaccine (Bart et al. 2014)
*Clostridium difficile*	Expansion	35 Y	Population expansion coincides with the first reports of hospital outbreaks (He et al. 2010). Recombination tracts removed
*Escherichia coli*	Expansion	140 MY	A population bottleneck had a founding effect by purging diversity and leading to the formation of the extant major groups of *E. coli* (Wirth et al. 2006). 50-fold population expansion in the last 5 MY. Mentions the caveat of recombination
*Legionella pneumophila*	Expansion	20 Y	Correlation between population and reported number of clinical cases (Sanchez-Buso et al. 2014). Recombination tracts removed
*Moraxella catarrhalis*	Expansion	50 MY	The populations of antibiotic resistant isolates expand faster than those of sensitive bacteria (Wirth et al. 2007). Recombination tracts removed
*Mycobacterium tuberculosis*	All expansion	70 KY, 6.6 KY, 40Y	(1) Concludes about a parallel evolution between human (mitochondria) and this clade’s *N*_e_ caused by a tight host-parasite association (Comas et al. 2013). (2) One expansion is associated with the industrial revolution, another with the first world war, and a recent contraction is associated with the introduction of antibiotherapy (Merker et al. 2015). (3) Expansion is associated with acquisition of multi-drug resistance (Eldholm et al. 2015)
*Mycoplasma gallisepticum*	Expansion	17 Y	Population expansion (Delaney et al. 2012)
*Neisseria gonorrhoeae*	Expansion, contraction	40 Y^a^, 120 Y	(1) Population expansion measured in housekeeping functions parallels the number of clinical cases, but not when measured in an antibiotic resistance gene, suggesting it has been subject to positive selection. Results could be used in managing resistance (Tazi et al. 2010). Found no recombination events in the set. (2) Suggests that demographic changes are associated with selective sweeps caused by antibiotic resistance, crack epidemics and urban-planning. *N*_e_ decrease associated with 5× decrease in the prevalence of this obligatory human pathogen (Perez-Losada et al. 2007). Recombination tracts were removed
*Pseudomonas aeruginosa*	Expansion	0.005/nt^b^	Assigns the presence of a recent selective sweep (Guttman et al. 2008)
*Pseudomonas fluorescens*	Stable	0.07/nt^b^	Suggests ancient rapid growth followed by stabilization, but very close strains are absent (Guttman et al. 2008)
*Pseudomonas syringae*	Stable	0.1/nt^b^	Suggests it is an endemic pathogen (Sarkar and Guttman 2004)
*Salmonella enterica* serovar Paratyphi A	Expansion	450 Y	Population contraction associated with the introduction of antibiotics, followed by expansion that would be associated with environmental changes (Zhou et al. 2014). Recombination tracts removed
*Salmonella enterica* serovar Typhi	All expansion	10–71 KY, 25 Y	(1) Steady increase in population size in the last 3,000 years. Recombinant SNPs removed and strong selection checked (Roumagnac et al. 2006). (2) Expansion is consistent with epidemiological data reporting drug-resistant isolates. Recombinant regions removed (Wong et al. 2015)
*Shigella sonnei*	Stable	500 Y	The population size was found to be constant through time (Holt et al. 2012)
*Staphylococcus aureus*	Expansion	20 Y, 50 Y, 30 Y	(1) Rampant expansion might have followed trans-Atlantic spread (Nubel et al. 2010). (2) Phylodynamics analysis used to estimate epidemiological parameters such as the potential reproductive number. No signs of recombination identified (Prosperi et al. 2013). (3) Fit between demographic expansion and the epidemiology of the CC80 clone (Stegger et al. 2014)
*Streptococcus pneumoniae*	Contraction	15 Y	Population expansion and then contraction fits the observed number of clinical cases (Croucher et al. 2014). Recombination tracts removed
*Streptococcus pyogenes*	Expansion	80 Y	Associates population expansion with the acquisition of super-antigens (Davies et al. 2015). Recombination tracts removed
*Streptococcus suis*	Expansion	90 Y	Correlates population expansion with the introduction of new methods used for improved pig genetics (Weinert et al. 2015). Recombination tracts removed
*Thiomonas spp*	Expansion	7 MY	The demographic history matches the glacial cycles (Liao and Huang 2012)
*Vibrio cholerae*	Expansion	3 Y	Association with the history of the progression of an epidemic (Azarian et al. 2014). Found no evidence for recombination

*Note*—We show the TMRCA, the conclusion of the work, and the authors' justifications of the results. Multiple studies published for a given species are indicated as multiple lines in the column TMRCA and by the respective numbers in the last column.

^a^TMRCA not indicated. The value indicates the span of the *X*-axis on the skyline plot.

^b^Studies did not perform time calibration and present only the number of mutations per site.

Are all bacterial populations expanding? Researchers might focus preferentially on expanding bacterial populations, for example, recent epidemic clones, thus producing an ascertainment bias towards population expansion. Also, human populations have been growing exponentially and human-specific pathogens might have followed similar trends. However, a number of arguments cast doubt on these results. (1) The prevalence of bacterial pathogens (the majority of species in table 1) has decreased in the last century as the result of hygiene and the use of antibiotics (Cohen 2000). (2) Most of the remaining species in table 1 are commensals associated with multiple hosts (eventually including some nosocomials), or free-living bacteria for which human population growth might be of little relevance (especially since it is associated with decrease in the population of closely related animals that are often within the commensal host range). For example, *E. coli* is associated with most warm-blooded and some cold-blooded animals (Tenaillon et al. 2010), *Moraxella* was until recently regarded exclusively as a commensal of animals (Brenner et al. 2005), and *Thiomonas* spp*.* are free-living bacteria inhabiting extreme environments (Liao and Huang 2012). (3) The majority of the studies in table 1 have not checked for the assumptions of the standard neutral model, and those that did, only checked for the presence of recombination. Very few studies have used SFS to infer demographic changes in bacterial populations. While several of these works obtained SFS compatible with recent demographic expansions, they also showed that distortions in the SFS were partially caused by purifying selection (Cornejo et al. 2013; Pepperell et al. 2013; Touchon et al. 2014). These arguments led us to study the effects of violations of the assumptions of the standard neutral model in the inference of bacterial demography.

### The Effect of Recombination

**Fig. 1.**
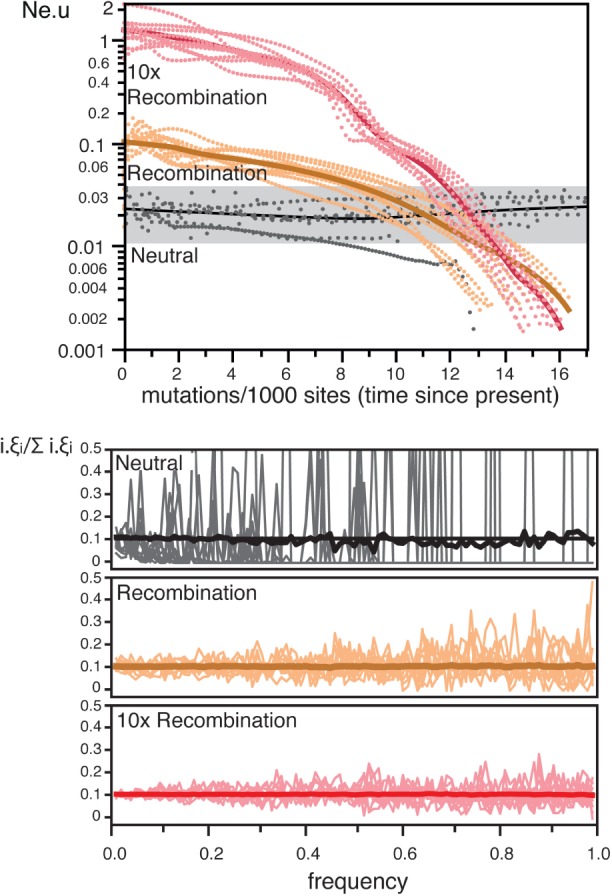
The effect of recombination on skyline plots and SFS. The simulations used the *E. coli* population parameters (*Recombination*), ten times higher recombination rates (*10× Recombination*), or no recombination (*Neutral*). *Top* The simulations in the skyline plots are represented as dotted lines. The thick lines represent the smooth kernel fit (resp. *R*^2 ^=^ ^0.81, *R*^2 ^=^ ^0.87, and *R*^2 ^=^ ^0.38). *Bottom*. SFS (distribution of the frequencies of all nucleotide polymorphisms in the sample) for each condition. The thick line indicates the average SFS over 1,000 replicates whereas the thin shaded lines are the observed SFS for ten random replicates. All SFS were transformed and normalized (see section “Methods”). Colors match the same datasets in both plots.

**Fig. 2.**
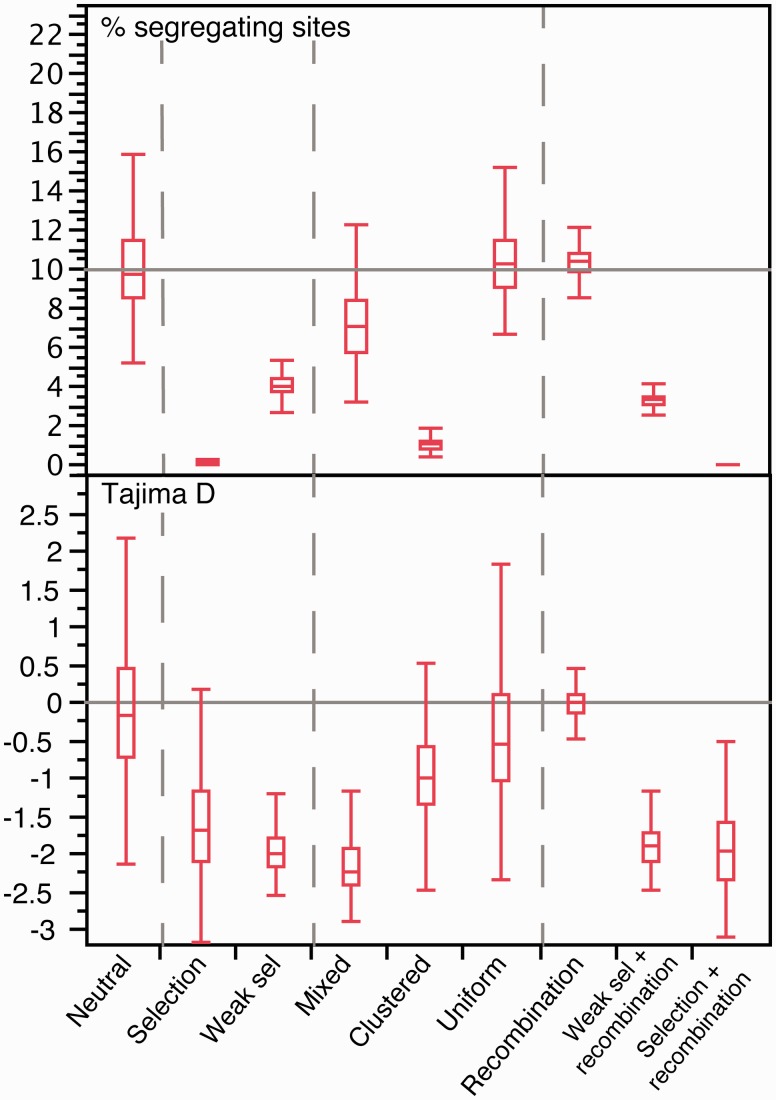
Distribution of the number of segregating sites and Tajima *D* values in each set of 1,000 simulations. The gray line in the top panel corresponds to the expected number of segregating sites under the standard neutral model: $\pi \text{=}\theta \text{.}L\text{.}a_{n}$ where $a_{n}=\sum_{1}^{n-1}\frac{1}{i}$. Here, θ=0.02, L=20,000, and n=100. The gray line in the bottom panel corresponds to the expected Tajima *D* under the neutral model (*D* = 0).

We made forward population genetics simulations of a locus of 20 kb with gene conversion and constant population size (see section “Methods”). Hence, deviations from the expectations of the neutral coalescent in the simulations were necessarily caused by recombination, not demography. The parameters for the simulations were taken from the literature for the model bacterium *E. coli* (table 2). Several studies estimated the rate of recombination over mutation in *E. coli* (reviewed in Bobay et al. 2015). We used an estimate based on the analysis of complete genomes (Touchon et al. 2009), which is among the lowest proposed and might therefore be conservative. The sequences resulting from our simulations were used to obtain skyline plots with BEAST (Drummond and Rambaut 2007). Our results show that even the moderate recombination rate observed in *E. coli*, leads to skyline plots with increasing values of *N*_e_.*u* for recent dates (fig. 1). This could be spuriously interpreted as an indication of population expansion. Simulations using ten times larger recombination rates (as observed in highly recombining bacteria), showed even stronger distortions in the skyline plots. Expectedly, recombination had no effect on the number of segregating sites (see *Recombination* in fig. 2), and lowered the variance, but did not affect the average, of the genome-wide average SFS (fig. 1). Consequently, recombination had no effect on the average estimate of Tajima *D* (although for a single locus see Thornton 2005).

**Table 2.** Parameters for *E. coli* Populations Used in the Simulations.

Parameter	Value	Reference
Effective population size (*N*_e_)	1.8 × 10^8^	Hartl et al. (1994)
Genomic adaptive mutation rate	1 × 10^−5^	Perfeito et al. (2007)
Genomic deleterious mutation rate	2 × 10^−4^	Kibota and Lynch (1996)
Average value of *s*^a^	±7 × 10^−3^	Perfeito et al. (2007) and Gallet et al. (2012)
Mutation rate per generation (u)	8.9 × 10^−11^	Wielgoss et al. (2011)
Genome size (nt)	5 × 10^6^	Touchon et al. (2009)
Recombination/mutation rate	1	Touchon et al. (2009)
Size of recombination tracts	542	Didelot et al. (2012)
SNPs recombination/mutation	2.5	Touchon et al. (2009)
Weak selection (*N*_e_.*s*)	5	
Strong recombination/mutation rate	10	

^a^The absolute values of *s* for adaptive and deleterious mutations being in the same order of magnitude we used an average for both.

**Fig. 3.**
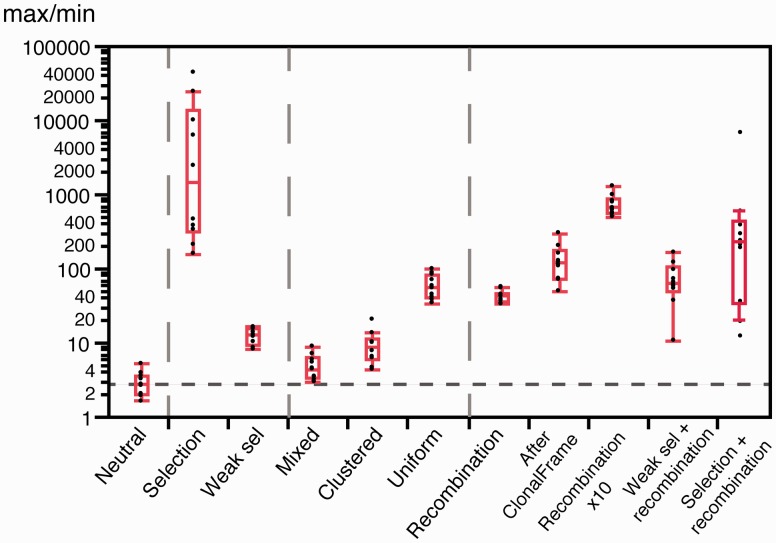
Boxplots of the ratios between the maximal and minimal *N*_e_.*u* values for skyline plots (ten simulations each), across the different types of simulations. All other categories were significantly different from *Neutral* (all *P* < 0.01 Wilcoxon tests, except the comparison between *Neutral* and *Mixed*, *P* = 0.0102, same test).

We then tested if state-of-the-art methods aiming at producing “recombination free” phylogenetic trees could produce unbiased skyline plots. We analyzed ten simulations with ClonalFrame to obtain a matrix of distances between individuals purged from recombination (Didelot and Falush 2007). We used these matrices to infer phylogenies and these phylogenies to compute skyline plots. The latter showed very clear and systematic increase in the values of *N*_e_.*u* for recent times (supplementary fig. S1, Supplementary Material online). The average amplitude in *N*_e_.*u* (measured as the ratio between the maximal and the minimal value) was three times higher than the one obtained without the use of ClonalFrame, that is, with the primary data (see *After ClonalFrame* in fig. 3). This suggests that ClonalFrame distance matrices are skewed so that the trees inferred from them have internal branches more affected by the removal of recombination than the external branches. These results are in line with a previous study showing that removing homoplasies in recombined sequences worsens the distortions in skyline plots (Hedge and Wilson 2014). Hence, trying to remove polymorphism caused by recombination may aggravate the biases of demographic studies using skyline plots.

### The Effect of Selection

**Fig. 4.**
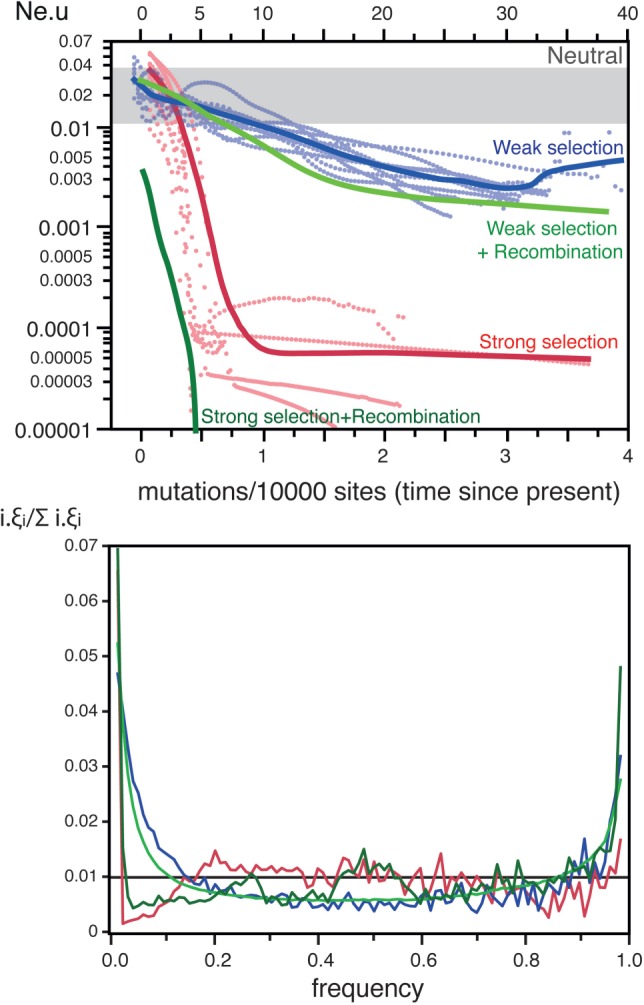
The effect of selection on ten skyline plots (top) and 1,000 SFS (bottom). *Top* The simulations were represented as dotted lines. The thick lines represent the smooth kernel fit for strong and weak selection (resp. *R*^2 ^=^ ^0.78, *R*^2 ^=^ ^0.79). For the analysis of selection and recombination only the kernel fits are indicated (*R*^2 ^=^ ^0.80). The grey box indicates the range of variation of the *Neutral* simulations in figure 1. *Bottom* The thick lines represent the average SFS over 1,000 simulations. In all SFS plots, the horizontal black line indicates the neutral expectation. Colors match the same datasets in both plots.

Experimental works indicate that >45% of the mutations are deleterious (Kibota and Lynch 1996), and >2% are adaptive (Perfeito et al. 2007) in *E. coli*. The effective population size of the species is estimated at >10^8^ (Hartl et al. 1994; Lynch 2006). The average selective effects of mutations in *E. coli* are much larger than the inverse of the effective population size (table 2), which implies that their fate is mostly driven by selection (Kimura 1983). Our simulations using these parameters resulted in very strong distortions in the skyline plots, showing higher *N*_e_.*u* values for recent dates (see *Selection* in fig. 3). These patterns might have been interpreted as population expansions if the effect of selection had been ignored. Under strong selection, diversity is constantly being purged and swept away by recurrent selective sweeps. Accordingly, the fraction of segregating sites in these simulations was only ∼0.16%, to be compared with ∼10% for the neutral simulations (see *Selection* in fig. 2). The effect of strong selection was also apparent in the SFS, where extremely rare and frequent alleles were in large excess (fig. 4), presumably due to the selective sweeps caused by beneficial mutations. This resulted in negative values of Tajima *D* (fig. 2).

Some of the species listed in table 1 have narrow host ranges and might have much smaller *N*_e_ than *E. coli*. We therefore made simulations using parameters corresponding to populations with *N*_e_.*s* = ±5 (*s* being the average selection coefficient on sites under selection) and a distribution of the frequency of sites under selection similar to *E. coli*. If these species have similar distributions of selective effects as those used *for E. coli* (*i.e.*, similar *s*), this value corresponds to *N*_e_ close to 1,000 (five orders of magnitude lower than *E. coli*). One should note that even bacteria obligatorily associated with humans are thought to have higher absolute values of *N*_e_ or *N*_e_.*s*, for example, the *N*_e_ of *Neisseria meningitidis* was estimated at 10^5^ (Treangen et al. 2008), and the average nonsynonymous values of *N*_e_.*s* were estimated at −5 for *M.* *tuberculosis* (Pepperell et al. 2013) and at −17 for *Streptococcus mutans* (Cornejo et al. 2013). As expected, simulations incorporating such weak selection showed patterns much less extreme than those obtained under strong selection. For example, the average fraction of segregating sites in the former was ∼4%, less than half of the neutral expectation but over two orders of magnitude more than under strong selection (see *Weak sel* in fig. 2). The skyline plots and the SFS under weak selection also showed less striking distortions (see *Weak sel* in figs. 3 and 4). Nevertheless, deviations from the expectation under neutral evolution were still very important in both analyses (negative Tajima *D*, fig. 2). These are likely to be caused by low-frequency segregating mildly deleterious mutations and by the selective sweeps caused by beneficial mutations. Hence, selection affects the inference of demography even when the values of *N*_e_ are uncharacteristically low for bacterial populations.

In our previous simulations, we have included positive and purifying selection. We therefore assessed the separate impact of each of these components of the evolutionary process on the skyline plots and on the SFS. For this we made simulations with just either positive or purifying selection. The effect of strong selection on skyline plots and SFS was caused exclusively by positive selection (supplementary fig. S2, Supplementary Material online). Accordingly, the SFS for strong purifying selection shows no excess of rare or frequent variants. This is because of the extremely rapid purge of deleterious mutations of strong effect. On the other hand, the significant effect of weak selection on the skyline plots and SFS is caused by both purifying and positive selection (supplementary fig. S3, Supplementary Material online). The SFS and skyline plots of populations evolving under weak purifying selection show an excess of rare variants and an increase in *N*_e_.*u* for recent times (supplementary fig. S4, Supplementary Material online). This shows that when selection is very strong, only positive selection affects the reconstructed genealogies, whereas when selection is weaker, both positive and purifying selection affect the reconstructed genealogies (and thus the skyline plot).

We then simulated the joint effects of selection and recombination on the reconstructed genealogies to check if recombination might moderate the effects of selection (fig. 4). The joint effect of recombination and selection (weak or strong) on the skyline plots was noticeable, that is, led to even stronger distortions in the plots, than the independent effects of each taken separately (*P* < 0.0001, Wilcoxon test). The SFS with selection and recombination were not appreciably different from the ones with selection under no recombination (compare the pairs of lines in the SFS of fig. 4). As a result, Tajima *D* is negative whenever there is selection, that is, with or without recombination (fig. 2). These results show that one cannot ignore the effect of selection on the analyses of bacterial demography.

### The Effect of Sampling Bias

**Fig. 5.**
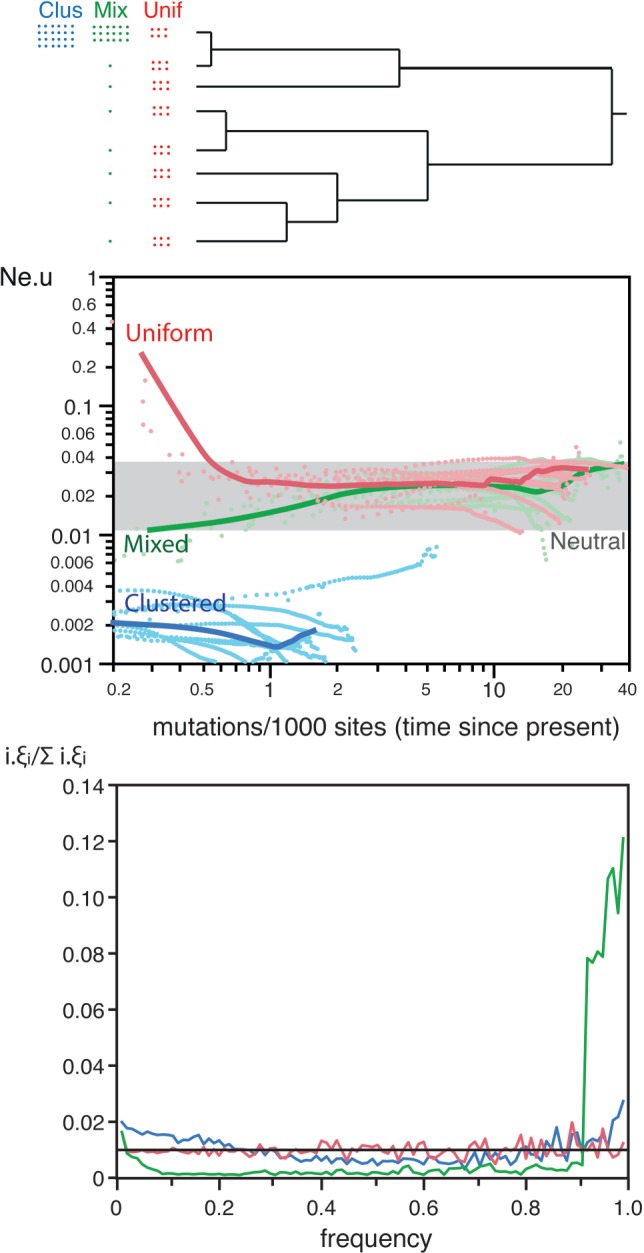
Analysis of three types of sampling biases. *Top* Schematic representation of the different types of sampling biases in a species tree (see section “Methods” for a precise definition). *Center* Skyline plots for each set of ten simulations. The dotted lines represent the simulations. The thick line represents the smooth kernel fit (resp. Clustered *R*^2 ^=^ ^0.63, Uniform *R*^2 ^=^ ^0.86, Mixed *R*^2 ^=^ ^0.40). The grey box indicates the range of variation of the *Neutral* simulations in figure 1. See supplementary figure S5, Supplementary Material online for a zoom for values of clustered bias close to zero. *Bottom* Average SFS for the three datasets (1,000 simulations for each). Colors match the same datasets in both plots.

We simulated three types of typical sampling biases in the study of microbial population genetics. In these simulations, there were no changes in population size, no selection, and no recombination. We simulated sampling biases by clustering the final individuals evolved in the simulations in groups using sequence similarity and then sampling these groups in different ways (see section “Methods”). The results showed that different types of sampling bias affect in very diverse ways the shape of the tree and of the SFS, and thus the inference of demographic changes (fig. 5).

The sampling of a single group (clustered sampling), resulted in skyline plots with lower average values of *N*_e_.*u*, as expected, and a peak of high *N*_e_.*u* for times very close to the present (see supplementary fig. S5, Supplementary Material online, for the values close to 0). The amplitudes of *N*_e_.*u* values were on average three times larger than those of neutral populations (*Clustered* in fig. 3). The simulations also showed slight over-representation of rare and frequent variants in the SFS. Clustered sampling produced alignments with far fewer (approximately ten times) segregating sites than the neutral simulations (*Clustered* in fig. 2). Hence, sampling a sub-population produces patterns akin to very recent population size expansions.

We simulated uniform sampling by re-sampling the same number of individuals in each group. This led to skyline plots with increasing values of *N*_e_.*u* for recent dates (fig. 5). In fact, this sampling bias resulted in reconstructed genealogies with fewer than expected short terminal branches, which is akin to the effect produced by strong population expansion. The consequent distortion of the reconstructed genealogies can be extremely important since these skyline plots had *N*_e_.*u* amplitudes >100 times higher than those found on neutral populations (*Uniform* in fig. 3). On the other hand, uniform sampling had essentially no effect on the SFS (fig. 5).

Mixed sampling bias was simulated by retrieving 91 individuals from one group and one from each of the remaining nine groups. These samples showed complex skyline plots, with initially increasing *N*_e_.*u* values followed by a sharp decrease for very recent dates (fig. 5). The SFS showed striking over-abundance of very frequent variants, some over-representation of rare variants and nearly no variants of intermediate frequency. This was associated with a negative Tajima *D* (*Mixed* in fig. 2). This pattern is the joint effect of the excess of very small external branches in the highly sampled group and the long internal branches linking the remaining groups in the reconstructed genealogy.

### Joint Effects of Selection, Recombination, and Sampling Bias

**Fig. 6.**
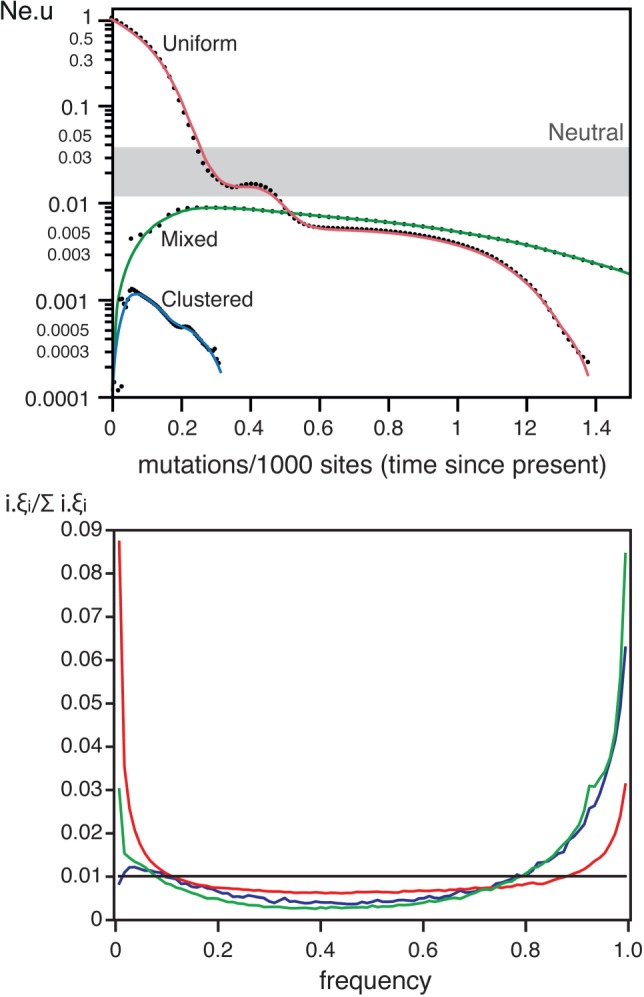
*Top* Skyline plots for clustered, uniform and mixed sampling on simulations with weak selection and recombination (each point is an average of the ten simulations). The grey box indicates the range of variation of the *Neutral* simulations in figure 1. *Bottom* Average SFS for the same three datasets (1,000 simulations). Colors match the same datasets in both plots.

We then studied the joint effect of sampling biases, recombination, and weak selection on skyline plots and SFS (as shown before, strong selection rapidly erases genetic diversity in the simulations). The increase in *N*_e_.*u* values in skyline plots inferred under uniform sampling bias was highly amplified when weak selection and recombination were also present, rising by almost four orders of magnitude (fig. 6). The SFS of these simulations showed a large excess of rare variants and a small excess of very frequent ones.

Clustered sampling of populations enduring recombination and weak selection resulted in skyline plots with a rapid increase in *N*_e_.*u*, which then rapidly dropped to values very close to the initial ones. This process mimics initial strong population expansion, followed by very recent strong population contraction. The SFS showed a slight excess of rare variants and a large excess of frequent ones.

Finally, the skyline plots of simulations with mixed sampling, recombination, and weak selection showed a steady increase in *N*_e_.*u* and then a sharp decrease near the present. These patterns are also akin to the effects caused by ancient population expansions and recent population contractions. The SFS of these simulations showed an excess of both rare and frequent variants, with few intermediate values.

### Analysis of the *E.* *coli* Core Genome

**Fig. 7.**
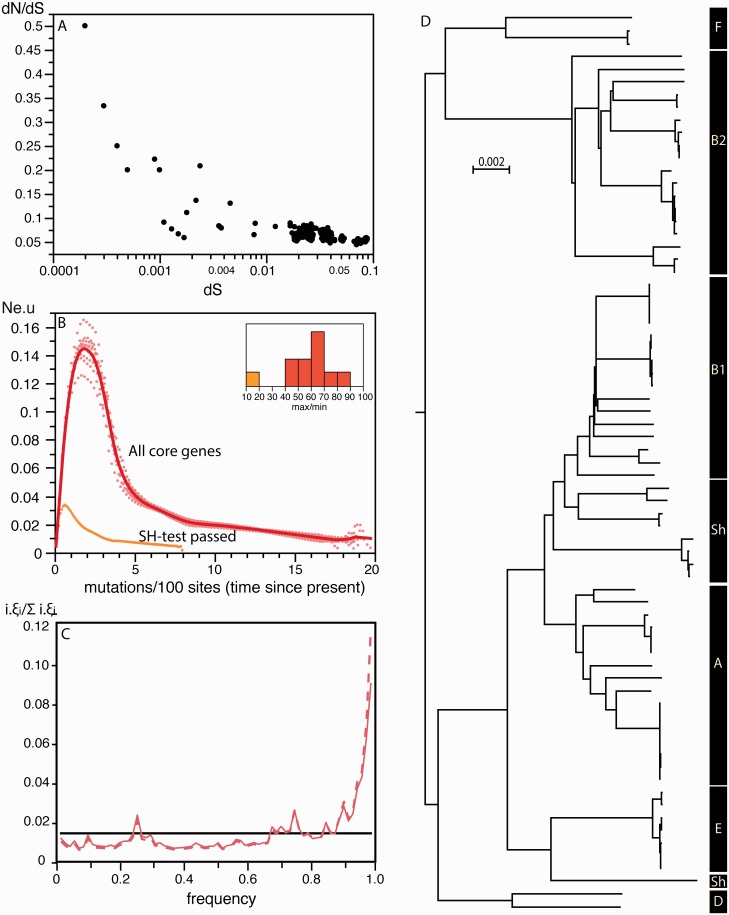
Analysis of the core genome of *E. coli.* (*A*) Values of *dN*/*dS* versus *dS*. Each point represents a comparison between two strains using the concatenate of alignments of genes of the core genome. (*B*) Skyline plot. We made ten analyses of the dataset by randomly sampling each time a tenth of the core genome. The orange line represents the skyline of the concatenate of genes with reconstructed genealogies not significantly different from those of the core genome (passed the SH test at *P* < 0.01). The inset represents the ratio between the maximum and minimum values of *N*_e_.*u* for the 11 skyline plots (10 with the 1/10th samples of the core genome and one with the analysis of the concatenate of genes passing the SH-test). (*C*) The observed SFS is indicated in dashed red line, the corrected SFS (with Kimura’s two-parameter model) is indicated in solid red line. The horizontal black line indicates the neutral expectation. The corrected SFS with the JC69 model (not shown here) is similar to the SFS corrected with Kimura’s two-parameter model except for the last point, which is slightly higher. (*D*) *E. coli* distance-based phenetic tree with the major clades indicated on the right. A similar tree indicating all strains used in the analysis is in supplementary figure S6, Supplementary Material online.

The parameters of fitness effects used in the simulations were measured on *E. coli* in the laboratory. It might be argued that these parameters are not representative of the effects observed in structured locally adapted natural populations. To assess the imprint of natural selection in *E. coli* we built its core genome (see section “Methods”). The analysis of the polymorphism in the ∼1.3 million positions of the alignment of *E. coli* core genes, showed a pervasive pattern of purifying selection as expected from the simulations (fig. 7). Indeed, the ratio between the rates of nonsynonymous and synonymous substitutions (*dN*/*dS*) was significantly lower than one for all pairwise comparisons with sufficient polymorphism. Importantly, when *dS* was higher than 1/5,000 the value of *dN*/*dS* was always smaller than 0.5. Multi-locus sequence typing (MLST) analyses use ∼5 kb of sequenced data and thus only start becoming informative when there is more than one SNP per 5 kb. At this level of divergence, the values of *dN*/*dS* show that the distribution of polymorphism is already imprinted by natural selection, precluding the use of MLST to make demographic inferences using skyline plots.

We then made ten random samples of 10% of the core genome positions to produce ten skyline plots for *E. coli*. The results were highly concordant between samples, showing a pattern of increase in *N*_e_.*u* followed by a sudden drop for times closer to the present (fig. 7). The SFS of the *E. coli* core genome showed a strong over-representation of very frequent variants (fig. 7). We then restricted our analysis to genes of the core genome with individual phylogenies not significantly different from those of the concatenate of the core genes. We found that the topologies of the reconstructed trees of 1,146 of the 1,371 core genes were significantly different from the one of the core genome (*P* < 0.01, Shimodaira–Hasegawa [SH] test). This analysis confirmed that the vast majority of genes in the genome are significantly affected by recombination, in spite of the low estimated rate of recombination in *E. coli*. We used the remaining 225 genes to build a skyline plot. This showed qualitatively identical trends, but less striking variations (fig. 7B).

Together, these results are consistent with a mixture of strong purifying selection and recombination producing patterns akin to demographic expansion in *E. coli* skyline plots. The excess of high-frequency variants observed in the unfolded SFS might be due to hitchhiking effects, appearing under strong selection and recombination. However, one cannot exclude the possibility that part of this excess might result from misoriented polymorphisms (polymorphisms for which the ancestral allele was wrongly assigned) (Baudry and Depaulis 2003), since corrections tend to lower this excess (see section “Methods” and fig. 7C). Alternatively, a mixed sampling bias could produce a drop in *N*_e_.*u* for the most recent times in skyline plots and a large excess of high-frequency variants in SFS. To test this hypothesis we built a phenetic tree for *E. coli* using a distance-based method (to minimize reconstruction artifacts associated with recombination). The analysis of this tree does not support the existence of a very strong mixed sampling bias (fig. 7).

## Discussion

Recent advances in the analysis of genetic data using coalescent theory have the potential to unravel many novel aspects of microbial population genetics. The limitations of the underlying models are well known from the theoretical point of view (Frost et al. 2014). However, at the beginning of this work it was unclear if these limitations could compromise the use of such approaches to analyze bacterial data. Our study suggests that neglecting the effect of natural selection, recombination, and sampling biases may severely affect conclusions from phylodynamics analyses. These results are likely to be applicable to other phyla where these effects are important. An important effect that we have not quantified in this study concerns population structure, which tends to produce patterns akin to population contraction (Pannell 2003). Unfortunately, we could not study them due to current lack of modeling frameworks for simulating bacterial population structure. Previous studies have confirmed that animal population structure leads to distortions in skyline plots (Heller et al. 2013).

Some of the studies in table 1 tried to eliminate the effect of recombination by removing detectable recombination tracts from the analysis. Using ClonalFrame, we obtained even worst distortions in skyline plots. Similar results were previously found for the removal of homoplasies (Hedge and Wilson 2014). While we cannot offer a clear explanation for this observation, we presume it is caused by the removal of only certain specific types of recombination events (or polymorphism) from the data. Interestingly, the analysis of *E. coli* genomes suggests that removing all genes whose trees are incongruent with that of the core genome (SH test) attenuates the effect of recombination. The reasons for this, and the consequences of removing these sequences, will require further study. Yet, the relative apparent success of this method might just derive from the bias of the SH test toward removing the recombining genes producing genealogies incompatible with the average genealogy of the core genome (while leaving for further analysis those that are compatible with this genealogy). This is expected to decrease the bias toward higher coalescent rates closer to the TMRCA. Importantly, the expectation of the SFS is insensitive to the presence of recombination and can be used to analyze genomic data deeply imprinted by recombination.

Previous theoretical studies suggested that selection on mutations of mild deleterious effect might not distort genealogies. This might explain why none of the studies in table 1 assessed the effect of natural selection on demographic inference. Yet, using population genetics parameters of *E. coli*, and even using much smaller values for *N*_e_.*s*, we found striking distortions in skyline plots.

We observed very frequent selective sweeps in the simulations with the selection parameters from *E. coli*. It must be emphasized that the high genetic diversity of the *E. coli* core genome is not fully consistent with such a succession of sweeps. However, it could be compatible with frequent soft sweeps, as recently described in *E. coli* adaptation to the mouse gut (Barroso-Batista et al. 2014). It would also be compatible with sweeps associated with local adaptation of certain lineages (Cohan and Perry 2007), or negative-frequency-dependent selection (Takeuchi et al. 2015). Finally, the existence of abundant strongly adaptive mutations in *E. coli* is consistent with previous results showing that a large fraction of amino acid substitutions between the *E. coli* and *Salmonella* lineages have been fixed by positive selection (Charlesworth and Eyre-Walker 2006).

To benefit from the power of coalescent-based approaches, one must find ways of controlling the distortions produced by selection on reconstructed genealogies. Unfortunately, practical and efficient ways of using the coalescent with selection are not yet available. Meanwhile, some simple controls might allow to identify or even estimate the effect of selection on demographic inference. For example, synonymous and nonsynonymous changes are very differently affected by selection, in spite of codon usage (Sharp et al. 2010), and partitioning the data in these two categories could shed light on the effect of selection on skyline plots and SFS. Comparisons between highly expressed and weakly expressed genes may also be informative since the former endure more intense selection for both synonymous and nonsynonymous substitutions (Rocha and Danchin 2004). Very recent polymorphism is relatively less imprinted by selection (Ho et al. 2005; Rocha et al. 2006), and might produce less biased patterns in skyline plots. Interestingly, the only published skyline plots in table 1 showing population contractions were based on samples with very short TMRCA (table 1). Unfortunately, the analysis of *dN*/*dS* in *E. coli* shows that even the very recent polymorphism was affected by purifying selection (fig. 7). Skyline plots on larger time spans are even more imprinted by natural selection and interpretation purely in terms of demographic changes should not be made in the absence of control for natural selection.

Random sampling is a key underlying hypothesis of most statistical methods for the inference of demographic changes. However, funding agencies often stimulate researchers to focus on particular bacterial sub-populations of societal interest. This renders random sampling effectively impossible and might explain why surveys of microbial populations rarely explicit the statistical design of the sampling. As an example, despite the fact that *E. coli* is a commensal present in most warm-blooded animals, the vast majority of complete genomes available for this species are from strains pathogenic to humans. Since host-association, virulence, and antibiotic resistance vary between lineages of a species, over-sampling isolates of direct interest in terms of public health almost inevitably leads to statistical biases. Our results show that three common sampling strategies can severely bias the inference of demographic changes, especially in the presence of selection and recombination. Skyline plots studies of populations where these factors are important can exhibit almost any possible pattern of change.

The sampling of sub-groups of a population led to reconstructed genealogies suggesting recent population expansion. These results show that sub-trees of coalescent trees have distributions of coalescent rates different from those of the population tree. Hence, sampling a sub-population inevitably produces biased skyline plots. This brings to the fore the importance of precisely defining bacterial populations when inferring demographic changes using coalescent rates. The study of past demographic changes in microbial populations requires the use of adapted sampling techniques. Many such techniques have been developed in ecology (Young and Young 2013), even if their implementation poses technical challenges in microbiological research.

Many approaches alternative to skyline plots allow the inference of demographic changes. They all have specific advantages and disadvantages and their combination might facilitate the use of the available sequence data to make demographic inference. Lack of obvious neutral sites in bacteria renders difficult the establishment of demographic models independent of selection. Nevertheless, *dN*/*dS*-based approaches can be used to assess if natural selection has imprinted sequence data (although care must be taken to check if absence of evidence of selection is not due to lack of statistical power). Furthermore, the expectations of the SFS are insensitive to recombination and to uniform sampling when there is no selection or recombination. They are also less affected by differences in the intensity of natural selection, although in case of pervasive selection with recombination, the SFS shape will correspond to the predictions of multiple merger coalescent models (Tellier and Lemaire 2014). Therefore, joint analyses of skyline plots, detection of recombination, SFS (and derived statistics), *dN*/*dS*, and other population genetics methods are necessary to accurately infer changes in microbial demography.

## Methods

### Simulations

We made 1,000 simulations for each set of parameters. Simulations were done using SFS_code, which implements a generalized version of the Wright–Fisher forward population genetic model allowing finite-site mutation models with selection, recombination, and demography (Hernandez 2008). The typical simulation was done using a population of haploids with *N*_e_ = 1,000 individuals and one single genetic locus of 20,000 nucleotides. The length of the locus was chosen in order to be much larger than the average recombination tract in *E. coli* (∼542 nt) (Didelot et al. 2012). In simulations under selection and recombination, we increased the length of the locus to 200,000 nucleotides, to obtain a sufficient number of polymorphic sites for further analyses. For simplicity, all nucleotides were included at similar frequencies and the substitution model was set to JC69 (equal mutation rates between all pairs of nucleotides) (Jukes and Cantor 1969). We used a 3-point mass model for selection (including negative, positive, and null values for the selection coefficient) (table 2). Modeling positive and purifying selection as two exponential distributions provides qualitatively similar results (but often produced numerical instabilities). Recombination was introduced exclusively as gene conversion (no crossovers allowed) in populations simulated as diploids (due to the constraints of the software). In this case, only half of the loci were used (1,000). The simulations were done using population scaled parameters accounting for the *N*_e_ of *E. coli* (table 2). Under these conditions, the size of the population effectively simulated does not affect the outcome of the analysis (Hernandez et al. 2007). In all cases, except those concerning sampling biases, we took 100 individuals from each final simulated population for further analysis.

### Simulations of Biased Sampling

When analyzing biased sampling we took all 1,000 individuals from the final simulated populations. These sequences were used to build a distance matrix with FastTree v 2.1.7 using default parameters and the option-makematrix (Price et al. 2009). This distance matrix was then partitioned into clusters around medoids, a more robust version of *K*-means (Reynolds et al. 2006), using R. We simulated biased sampling of 100 individuals from the population in three ways. We simulated uniform distribution by picking one individual per cluster in an analysis where the population was clustered in 100 groups. We simulated mixed sampling bias by picking one individual per cluster for a total of ten individuals and then picking the remaining 90 individuals from one single cluster (analysis where the population was clustered in ten groups). We simulated clustered distribution by selecting all 100 individuals from a single cluster (analysis where the population was clustered in ten groups). It is important to note that a cluster obtained with this method may not exactly correspond to a monophyletic group as described in figure 5. The goal of our approach was to mimic the typical identification of clusters of bacterial groups used to select strains for sequencing, which are based on relatively imprecise methods (MLST or PFGE).

### Analyses of Reconstructed Genealogies

We analyzed sequences using the generalized skyline plot model in BEAST with piecewise-linear modeling of the population size (skyline.popSize priors: initial = 3.2 × 10^−^^4^, upper = 100, lower = 0), using the HKY model (the mutation model was parameterized so that its stationary frequencies were the empirical frequencies) (Hasegawa et al. 1985), setting a tight prior for *k* (lognormal, initial = 1, logMean = 0, Logstdev = 0.25), a strict molecular clock (as used in the simulations), and 30,000,000 iterations (sampling every 3,000 iterations). For simulations involving selection we made 300,000,000 iterations. The effective sample size (ESS) values were checked using Tracer and the runs were accepted when the ESS was higher than 200 for all parameters with eventual exception for some skyline.population parameters (as suggested by the manual of BEAST—[Drummond and Rambaut 2007]). Analyses resulting in poor ESS values were discarded and re-run. Tracer was used to compute all skyline plots except those made after the ClonalFrame analysis (see below). Given the computational cost of these analyses we only analyzed ten simulations per condition. However, the results were very consistent between simulations resulting in kernel fits with high *R*^2^ (see text).

### Analysis of the SFS

SFS were generated from random samples of 100 individuals. The mean SFS was calculated using 1,000 simulations. The exact ancestral state of each SNP was obtained using SFS_code. The SFS of the simulations were thus unfolded. For a better representation of the results, the SFS were transformed as follows. Let $\xi_{i}$ denote the number of polymorphic sites at frequency $\frac{i}{n}$ in the sample of size n. We plot ${\text{i.}\xi }_{i}$ for $i\;\in \left[1,n-1\right],\;$ normalized by its sum, which is an unbiased estimator of the (supposedly unknown) mutation rate, often noted θ under the standard neutral model. Thus, the transformed SFS has a flat expectation under the standard neutral model, due to the well-known fact that $E\left[\xi_{i}\right]\text{=}\frac{\theta }{i}$ .

For the analysis of *E. coli* data, the ancestral state is unknown and we used outgroup sequences. To correct for potential ancestral misorientations (i.e., when the nucleotide of the outgroup is erroneously inferred as the ancestral state), we calculated the probability of misorientations, using sites for which the outgroup nucleotide is different from the two nucleotides of the SNP (see Baudry and Depaulis 2003; Hernandez et al. 2007).

If q is the probability that the outgroup nucleotide is identical to the ancestral nucleotide, we have in expectation:

$$\xi_{k}^{\text{obs}}=\xi_{k}q+\xi_{n-k}\left(1-q\right)\;\text{for}\;k\in \left[1,n-1\right],$$

where $\xi_{k}^{obs}$ is the number of polymorphic sites at frequency $\frac{k}{n}$ before correction and $\xi_{k}$ the real value.

We denoted by *S* the event that a given site is segregating, and by *U* the event that it is segregating *and* the outgroup nucleotide is different from the two nucleotides of the SNP. On one hand, *P*(*U* | *S*) is easily estimated by the proportion *x* of sites that are segregating and yet have a different outgroup nucleotide. On the other hand, under the JC69 model of mutation, *P*(*U* ∩ *S*) = 2*q P*(*S*), neglecting the case when the ancestral nucleotide is different from the other three. Combining these two arguments we can estimate *q* by *x*/2.

Once *q* is estimated from the data, we can calculate the corrected values of the SFS:

$$\xi_{k}=\frac{\xi_{k}^{obs}-\xi_{n-k}^{obs}(1-q)}{2q-1}\;\text{for}\;k\in \left[1,n-1\right].$$

We estimated *q* with two corrections, depending on the mutation model. Under the JC69 model of mutation, *q* = 0.960. Under Kimura’s two parameters model (Kimura 1980), taking into account the transition and transversion rates, *q* = 0. 947 (Baudry and Depaulis 2003).

### ClonalFrame Analysis and Subsequent Skyline Plot

ClonalFrame was used with default parameters on the results of ten simulations with recombination, no selection and no sampling bias. All ClonalFrame outputs were imported in the ClonalFrame GUI (Didelot and Falush 2007). The convergence of MCMC traces was visually assessed. ClonalFrame outputs ultra-metric trees with multifurcations, but bifurcating trees are necessary to compute skyline plots. Hence, for each simulation, we exported the recombination-free distance matrix and used the R package *phangorn* to construct the UPGMA trees (Schliep 2011). We computed generalized skyline plots using the skyline function of the *ape* package (Paradis et al. 2004). The AIC criterion was applied to find the optimal *ϵ* spline parameter.

### Analysis of *E. coli* Genome

We downloaded from RefSeq in November 2013 (Tatusova et al. 2015) the 62 genomes of *E. coli*, the nine genomes of *Shigella* spp. (in fact *E. coli* strains—Ochman et al. 1983) and the genome of *E. fergusonii* (the outgroup). Pairs of orthologous genes between two genomes were defined as bi-directional best hits, with >80% similarity in protein sequence, <20% difference in gene size, present within similar genetic neighborhoods (see Touchon et al. 2009 for details). The list of the core genome was defined as the intersection of all lists of pairwise analyses and included 1,371 genes. Genes from the same family of the core genome were aligned in protein sequence using MUSCLE v3.8 (default parameters, Edgar 2004) and back translated to DNA. These alignments were concatenated, making a total of 1,349,016 positions. They were used to compute the pairwise values of *dS*, *dN* and *dN*/*dS* between *E. coli* genomes using codeML from PAML v4 (parameters: runmode = −2; CodonFreq = 2; clock = 0; model = 2) (Yang 2007). Comparisons between very closely related isolates (i.e., with no single synonymous or nonsynonymous substitution in the core genome) were discarded.

### SH Tests and Phenetic Tree

We built a phylogenetic tree of the core genome of *E. coli* using IQ-Tree (Nguyen et al. 2015) with the option to search for the best substitution model. The best model based on the BIC criterion was GTR + I + G4. For each gene we used IQ-Tree to make the SH test (1,000 replicates) using as a reference tree the core genome tree. The phenetic tree in figure 7 was built using BIONJ (Gascuel 1997) from a distance matrix computed using TreePuzzle with the model GTR + I+G4 (Schmidt et al. 2002).

## Supplementary Material

Supplementary figures S1–S6 are available at *Molecular Biology and Evolution* online (http://www.mbe.oxfordjournals.org/).

Supplementary Data
