# Supplementary material for: The Impact of Selection, Gene Conversion, and Biased Sampling on the Assessment of Microbial Demography
Source: Mol Biol Evol. 2016 Mar 1;33(7):1711–25. doi: 10.1093/molbev/msw048 (PMC4915353; doi:10.1093/molbev/msw048)
Supplement: Supplementary Data [file supp_33_7_1711__index.html]

The Impact of Selection, Gene Conversion, and Biased Sampling on the Assessment of Microbial Demography — The Impact of Selection, Gene Conversion, and Biased Sampling on the Assessment of Microbial Demography — Supplementary Data 

# The Impact of Selection, Gene Conversion, and Biased Sampling on the Assessment of Microbial Demography

## Supplementary Data

files

- Supplementary Data - pdf file
