# Supplementary material for: The Impact of Selection, Gene Conversion, and Biased Sampling on the Assessment of Microbial Demography
Source: Mol Biol Evol. 2016 Mar 1;33(7):1711–25. doi: 10.1093/molbev/msw048 (PMC4915353; doi:10.1093/molbev/msw048)
Supplement: Supplementary Data [file supp_msw048_Lapierre_etal_supmat.pdf]

Supplementary material for :

The impact of selection, gene conversion and biased sampling on the assessment of microbial demography

by

Marguerite Lapierre, Camille Blin, Amaury Lambert, Guillaume Achaz, Eduardo PC Rocha

Table of contents:

- Figure S1
- Figure S2
- Figure S3
- Figure S4
- Figure S5
- Figure S6

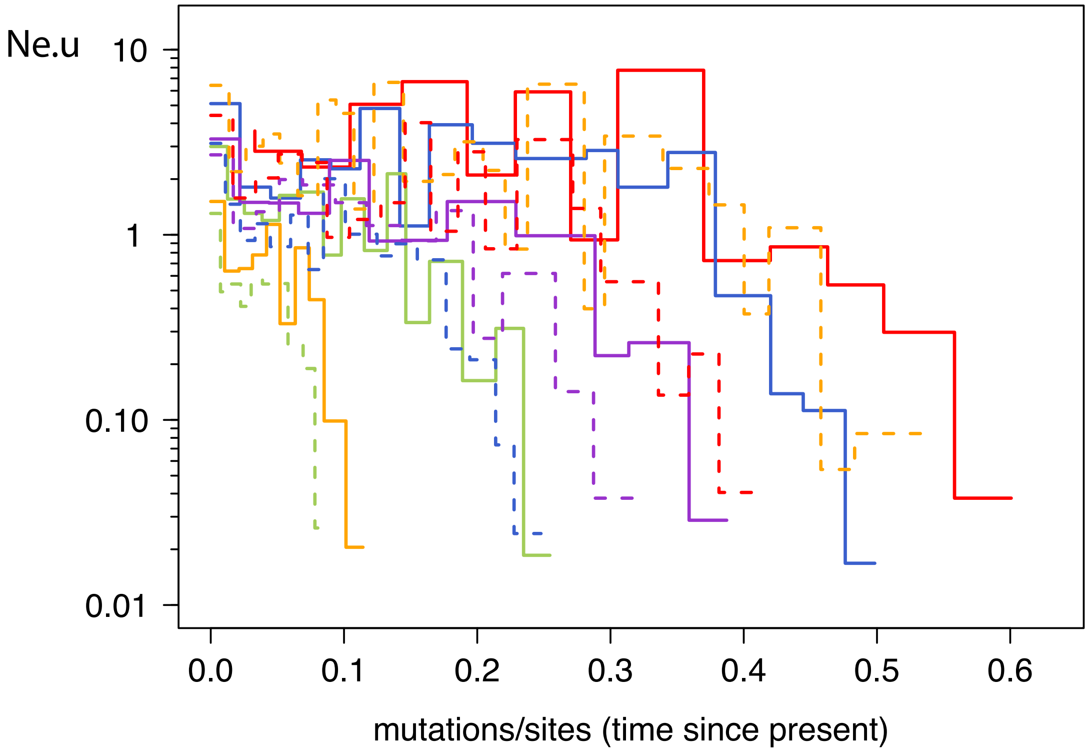

Figure S1- Skyline plots from the 10 simulations with recombination after analysis with ClonalFrame.

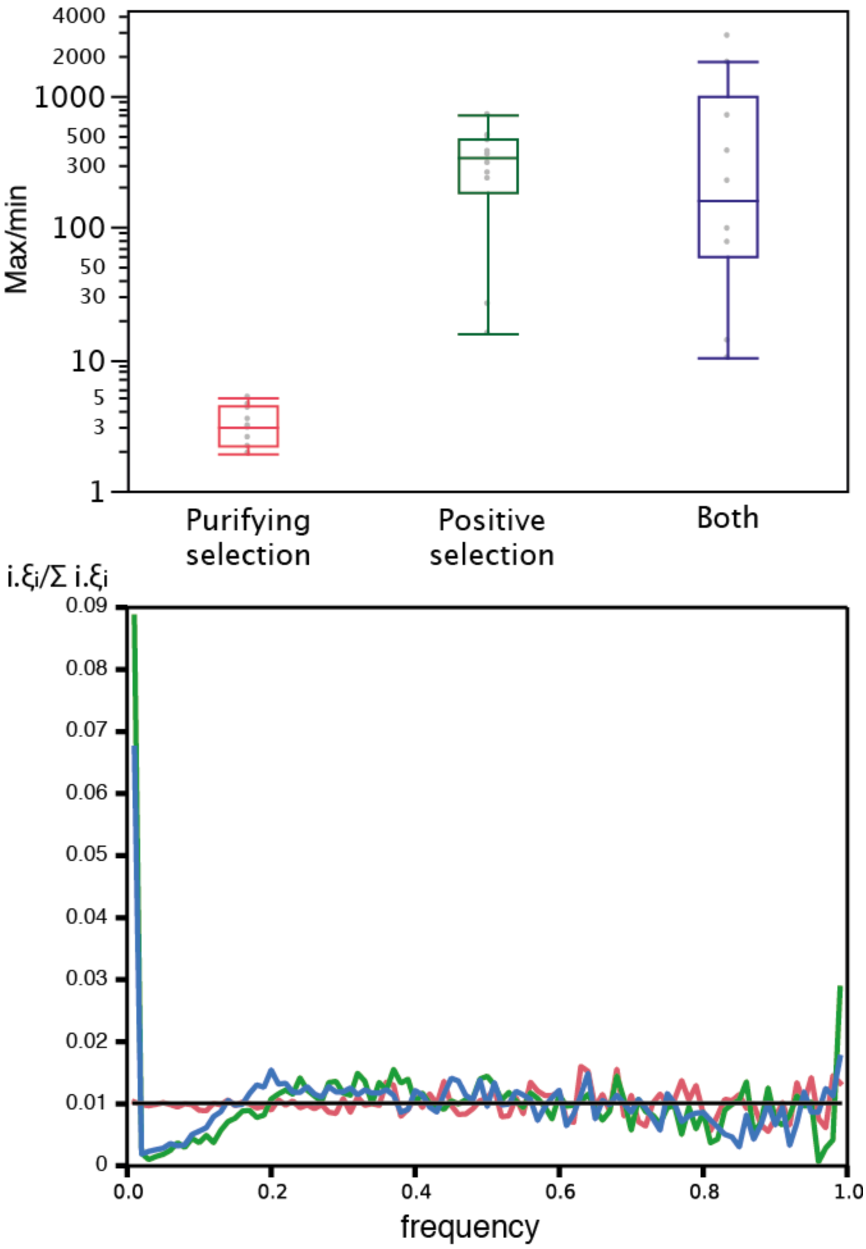

Figure S2 – Analysis of the components of simulations using selection. A. Boxplots of the ratios between the maximal and minimal Ne.u values for skyline plots, across the different types of simulations using the parameters for strong selection (10 simulations each). The hypothesis that the distributions are similar is rejected (P<0.001, Wilcoxon test), because of the purifying selection set, which is different from the others (P<0.001 same tests). B. SFS for the same conditions (1000 simulations each).

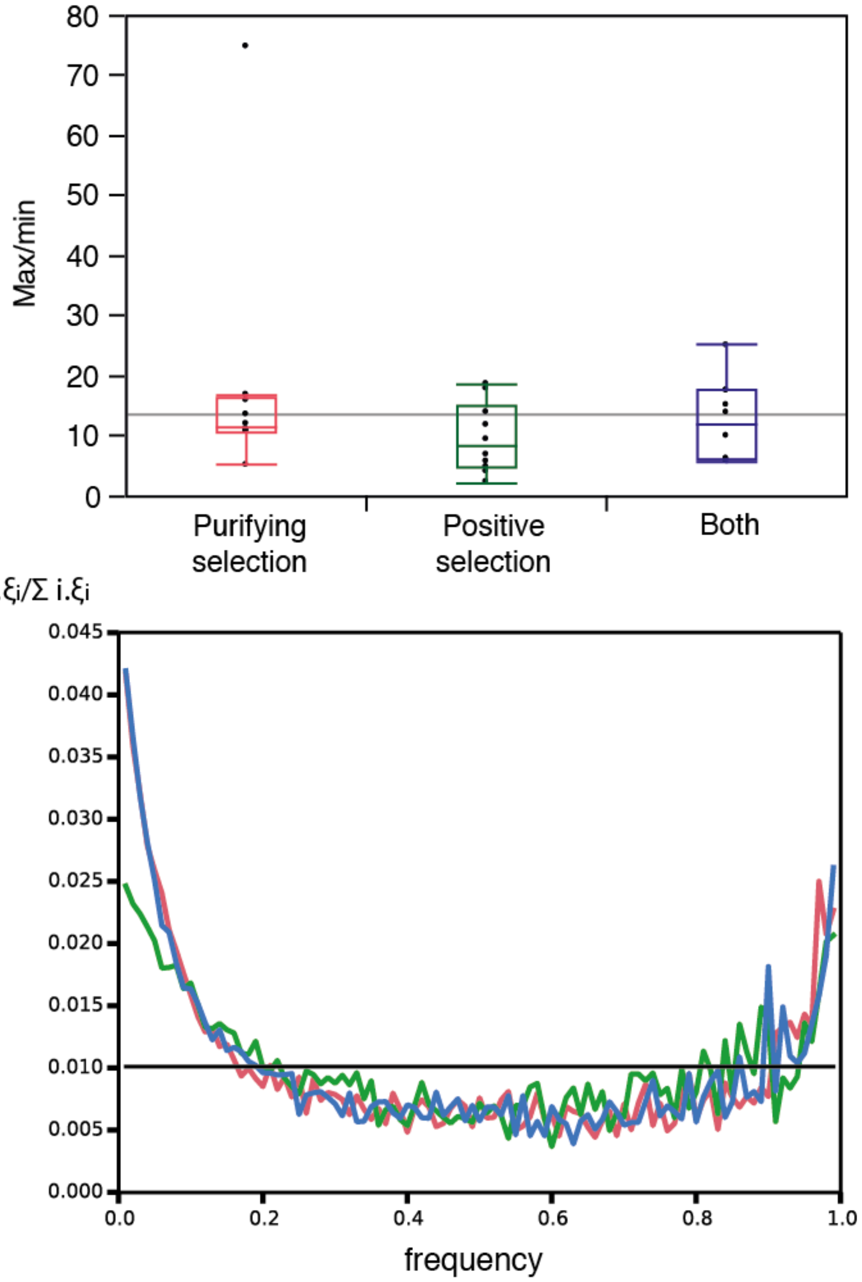

Figure S3 – Analysis of the components of weak selection simulations. A. Boxplots of the ratios between the maximal and minimal Ne.u values for skyline plots, across the different types of simulations using the parameters for weak selection (10 simulations each). The hypothesis that the distributions are similar is not rejected (P=0.4, Wilcoxon test). B. SFS for the same conditions (1000 simulations each).

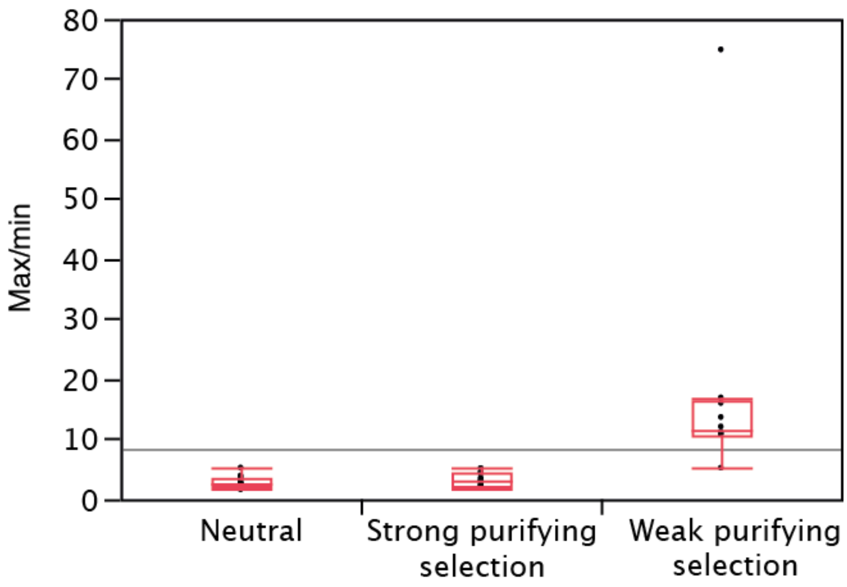

Figure S4 – Boxplots of the ratios between the maximal and minimal Ne.u values for skyline plots, across the different types of simulations using the parameters for no selection (neutral), strong purifying selection, and weak purifying selection. The hypothesis that the distributions are similar is rejected (P<0.001, Wilcoxon test).

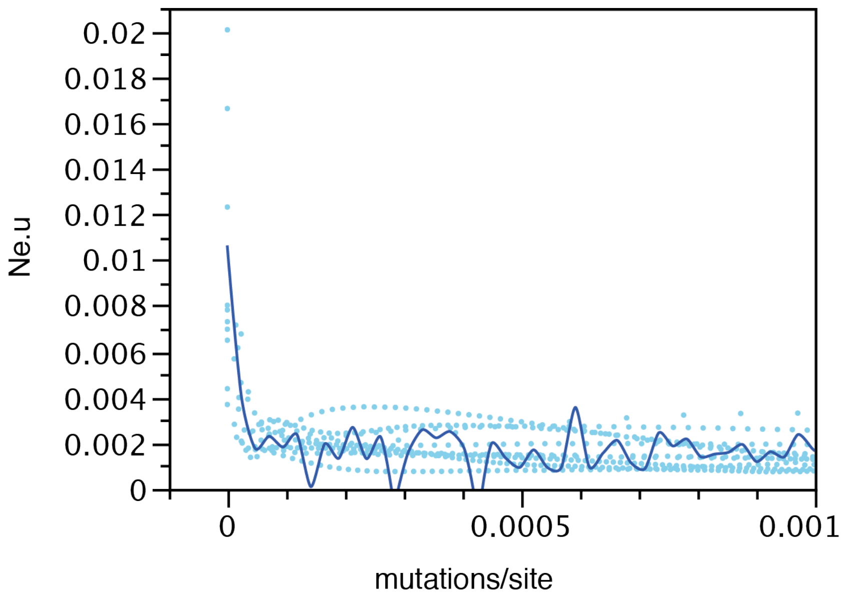

Figure S5 – Zoom of Figure 5 of the main text for the data using a clustered sampling bias. The figure in the main text lacks the values closer to zero (because they are log transformed). In this figure it can be seen that for values close to zero there is a systematic increase of Ne.u.

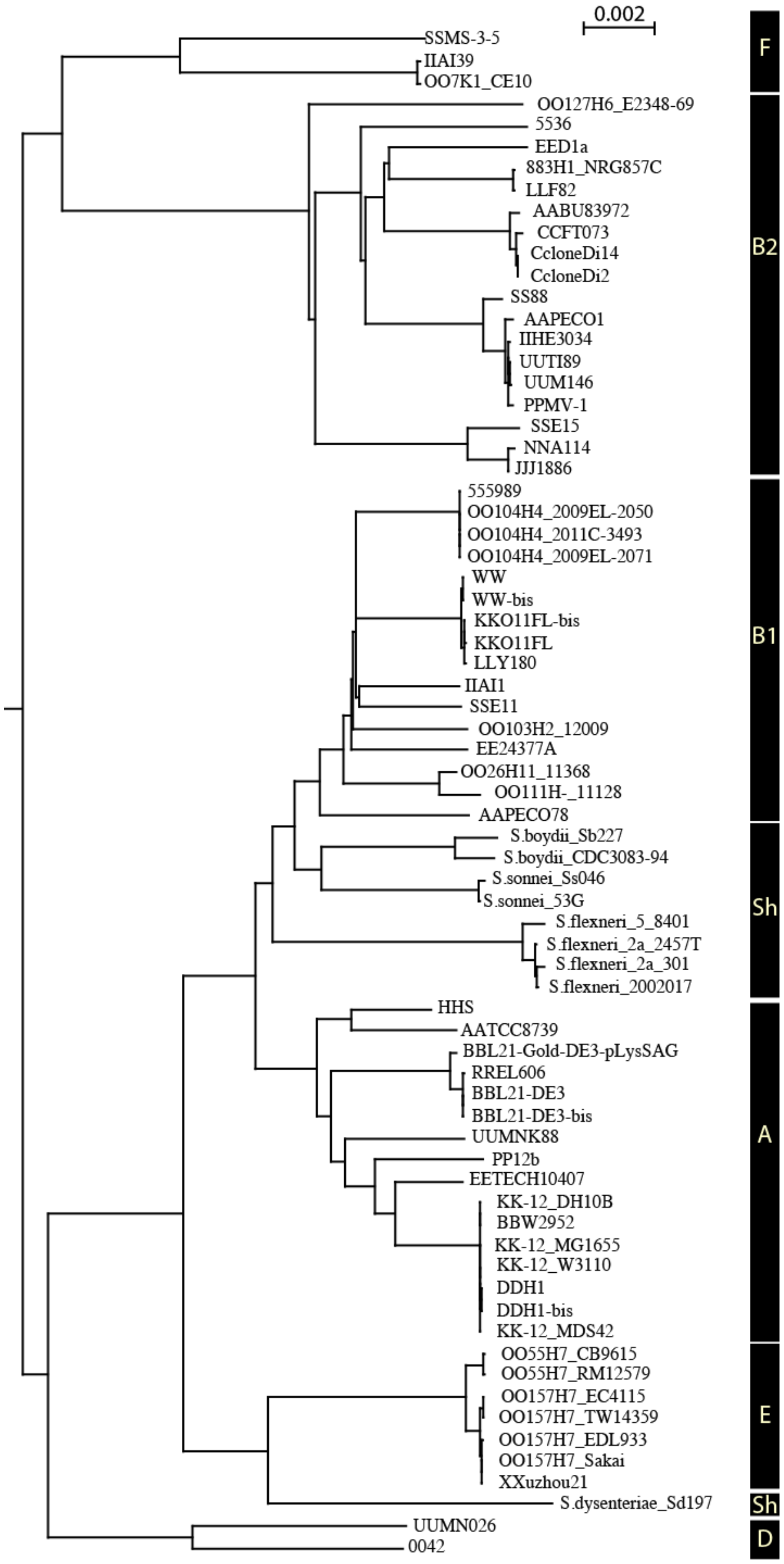

Figure S6- Neighbor-joining phylogenetic tree of *E. coli* with the indication of the major sub-clades on the right and the name of the strains used in the study.
